# Evaluation of the national roll-out of parenting programmes across England: the parenting early intervention programme (PEIP)

**DOI:** 10.1186/1471-2458-13-972

**Published:** 2013-10-19

**Authors:** Geoff Lindsay, Steve Strand

**Affiliations:** 1 Centre for Educational Development, Appraisal and Research (CEDAR), University of Warwick, Kirby Corner Road, Coventry CV4 7AL, UK; 2 Department for Education, University of Oxford, 15 Norham Gardens, Oxford OX2 6PY, USA

**Keywords:** Parenting programme, Evidence based practice, Effectiveness trial, National roll-out, Triple P, Incredible years, Strengthening families strengthening communities, FAST, Strengthening families programme 10-14

## Abstract

**Background:**

Evidence based parenting programmes can improve parenting skills and the behaviour of children exhibiting, or at risk of developing, antisocial behaviour. In order to develop a public policy for delivering these programmes it is necessary not only to demonstrate their efficacy through rigorous trials but also to determine that they can be rolled out on a large scale. The aim of the present study was to evaluate the UK government funded national implementation of its Parenting Early Intervention Programme, a national roll-out of parenting programmes for parents of children 8–13 years in all 152 local authorities (LAs) across England. Building upon our study of the Pathfinder (2006–08) implemented in 18 LAs. To the best of our knowledge this is the first comparative study of a national roll-out of parenting programmes and the first study of parents of children 8–13 years.

**Methods:**

The UK government funded English LAs to implement one or more of five evidence based programmes (later increased to eight): Triple P, Incredible Years, Strengthening Families Strengthening Communities, Families and Schools Together (FAST), and the Strengthening Families Programme (10–14). Parents completed measures of parenting style (laxness and over-reactivity), and mental well-being, and also child behaviour at three time points: pre- and post-course and again one year later.

**Results:**

6143 parents from 43 LAs were included in the study of whom 3325 provided post-test data and 1035 parents provided data at one-year follow up. There were significant improvements for each programme, with effect sizes (Cohen’s *d*) for the combined sample of 0.72 parenting laxness, 0.85 parenting over-reactivity, 0.79 parent mental well-being, and 0.45 for child conduct problems. These improvements were largely maintained one year later. All four programmes for which we had sufficient data for comparison were effective. There were generally larger effects on both parent and child measures for Triple P, but not all between programme comparisons were significant. Results for the targeted group of parents of children 8–13 years were very similar.

**Conclusions:**

Evidence-based parenting programmes can be rolled out effectively in community settings on a national scale. This study also demonstrates the impact of research on shaping government policy.

## Background

The implementation of parenting programmes has become an important part of the support provided by public health and education services to assist parents in bringing up their children. Evidence based parenting programmes provide a well-established means of increasing the prevalence of nurturing environments for children, both to promote positive behaviours and to prevent the development of behavioural, emotional and social difficulties (BESD) [1]. In terms of public policy, the issues include whether to support the implementation of parenting programmes and, if so, by what means. In particular, a key policy decision concerns whether to target specific groups at risk or to implement a universal intervention aimed to bring benefits to the whole population. The present paper examines the UK Government’s national implementation of a targeted initiative of national support for evidence based parenting programmes for children with, or at risk of developing, BESD, the Parenting Early Intervention Programme (PEIP). The PEIP was implemented in all 152 local authorities (LAs) throughout England (2008–11). The paper builds on our earlier report of the evaluation of the Pathfinder implemented in 18 LAs (2006–08) [2].

Evidence based practice is dependent upon several factors: i) that interventions are effective; ii) that a policy has been approved for implementation on a specified scale: local, regional or national; iii) that there are systems for ensuring that practitioners and policy makers concerned with implementation have the necessary skills and knowledge to interpret the evidence, and have necessary implementation skills; iv) a supportive context to host the implementation; v) recognition by policy makers of the potential benefits of addressing the identified problem balanced against cost and competing priorities; vi) the political will to implement this specific intervention, providing appropriate resources and support.

There is now a substantial evidence base for the efficacy of parenting programmes to address children’s BESD, which pose a substantial challenge to individual parents [3] and society as a whole [4]. Reviews have been conducted of specific programmes, such as the meta-analysis by Nowak and Heinrichs [5] of Triple P and systematic reviews of a range of parenting programmes [6-10]. Organisations have also developed criteria for determining which parenting programmes meet acceptable evidence standards for recommendation to practitioners, including the United Nations Office on Drugs and Crime (UNODC) [11] and the National Academy of Parenting Research in England [12].

In order that a public policy of implementing evidence based parenting programmes can be delivered successfully, several stages of development and evidence creation are necessary. First, the parenting programme must be based on sound theoretical underpinnings for which there is an existing evidence base [11]. Second, a programme must be created based on these theoretical principles and that evidence base, together with an effective framework for implementation. The latter should include systems of ensuring access to the target population, effective recruitment and retention. Third, initial studies will test out developments that will lead to the trial version of the programme. Fourth, an efficacy trial that meets accepted quality standards, for example those of the Society of Prevention Research [13], will be required to establish that the programme produces improvements in the target behaviours: improved parenting skills and improved child behaviour as the primary objectives. Other secondary impacts, such as improvements in parental mental well-being or reduction in depression, may also be included [14]. Furthermore, efficacy trials will be required to demonstrate that short term improvements from pre- to post-course are maintained over longer periods of time.

Fifth, independent efficacy studies are advised in order to demonstrate generalisability of effects beyond trials run by the programme originator. These are important as there is evidence that independent trials show much smaller, or even no effects, compared with trials by the originator [15]. Sixth, programme implementation must be examined by efficacy trials in real world, community settings through efficacy trials. Finally, there is benefit in studying whether the improvements shown in the efficacy trials, whether research- or community-based, are maintained when implementation is scaled up: an *effectiveness* trial. At this point the intensity of commitment, oversight and support provided by the originator and any dedicated research team, as in earlier levels of this process, is much reduced. Also, many more staff must be recruited and trained, and a larger organisation to manage and support the implementation is necessary. Nevertheless, this research and development phase is crucial to ensure that effects are maintained when the programme is implemented on the scale necessary for public health and education policy.

Successful scaling up of evidence based parenting programmes in community settings requires that the community services buy into the need for the programme and undertake necessary developments to implement it successfully [16]. A key factor is the context provided by the community services, which will influence the knowledge transfer process. Kothari and Armstrong [17] argue that traditional medical decision-making, focusing on health outcomes for individual patients and changes in specific clinical behaviours of the professional, is not appropriate for public policy settings. There has been relatively little research on this approach in public policy settings and limited evidence of its success [18]. Kothari and Armstrong argue for the importance of collaboration within and across organisations.

One important strategy to enhance knowledge transfer of evidence-based programmes is the delivery of training of the parenting group facilitators by the programme staff, either by the programme developers or by staff trained by the programme team. Some established parenting programmes, as used in the present study, have developed rigorous training systems which they deliver to organisations wanting to use their programmes. In addition, the programmes are manualised to have standard course design, content and procedures. Teaching and learning materials are also provided, including DVDs to provide examples for discussion and to support teaching parents, and parent homework materials to support consolidation of learning.

Scaling up may be implemented in different ways. For example, local agencies may determine to implement a parenting programme, or a superordinate agency may take a policy decision which is then implemented at local level. Olds [19] provides an example of the former. Following positive evidence of the Nurse-Family Partnership programme, Olds and colleagues set up the National Center for Children, Families and Communities, with the purpose of supporting the development with fidelity of implementation of the programme in new communities, with fidelity. Many evidence-based parenting programmes provide comparable approaches, limiting the implementation of the programme to those who have undergone specified training. In each case the programme organisations have put a high value on scientific integrity, with methods to optimise training (and hence determine which practitioners are able to implement the programme) and the fidelity of implementation. The PEIP was implemented following a policy driven decision at national level by the UK Government’s Department for Children, Schools and Families (DCSF) for England, now the Department for Education (DfE), to fund parenting programmes in all English LAs and to use the programmes’ personnel to implement training of group facilitators.

The DCSF developed its implementation of parenting programmes as part of a broader policy agenda seeking to address behavioural difficulties including delinquency in young people: The Respect Action Plan [20]. A three stage development took place, although this comprehensive sequential approach was not set out at the start. First, a review of the literature on parenting programmes was commissioned [9]. On the basis of this review the DCSF undertook an initial implementation of parenting programmes over 2 years in 18 of England’s 152 LAs: the Parenting Early Intervention Pathfinder. Funding of £7.6 million was allocated for LAs’ to develop an organisational infrastructure, to fund training of facilitators and to run parenting groups. Three parenting programmes were selected, on the basis of the review by Moran et al. [9]. Funding was provided for 18 LAs, six to implement each of the three programmes.

Our evaluation of the Pathfinder indicated that the three programmes had all been implemented effectively [2]. We also provided evidence for the process factors that were important in supporting successful implementation [21]. On the basis of this evidence, the UK government decided to increase the scale and fund all English higher tier LAs. The DCSF also decided to fund an evaluation of this national roll-out of evidence-based parenting programmes, in order to examine whether the effectiveness, demonstrated by the Pathfinder in 18 LAs, would be maintained when the delivery was scaled up to implement parenting programmes in all 152 LAs in England.

### Aims

The overall evaluation comprised a study of effectiveness of the parenting programmes and also a study of the process factors influencing the success of the implementation of the PEIP. This paper addresses the first purpose. The study reported here had four aims:

1. To evaluate whether the positive impacts of parenting programmes demonstrated in efficacy trials are replicated when the programmes are rolled out on a national scale.

2. To examine whether the positive benefits of the parenting programmes are sustained one year later.

3. To examine the relative effectiveness of the parenting programmes used in the national roll-out, in both the short term and one year later.

4. To examine the effects specifically for parents of children 8–13 years, and for their children.

## Methods

### Design

The Department for Children, Schools and Families (DCSF), later the Department for Education (DfE), allocated funding to all English LAs in three waves. The 18 LAs involved in the Pathfinder continued to receive funding (Wave 1). Wave 2 comprised 23 LAs whose funding began in 2008. The remaining 111 LAs started to receive their funding in 2009. In all cases funding continued until 31^st^ March 2011. Sums allocated were determined by the DCSF and varied, for example, by size of LA population. Allocations in 2009–10 ranged from £25,000 to £260,000 per LA, with a total budget of £25.8 million.

The PEIP was set up with designated finance that was ring-fenced and could only be used to support the implementation of parenting programmes specified by the DfE. These were accredited following reviews undertaken by the National Academy of Parenting Practitioners (NAPP). Initially, five programmes were accredited. These comprised the three implemented during the Pathfinder: Triple P [22], Incredible Years [23] and Strengthening Families Strengthening Communities (SFSC) [24] together with Families and Schools Together (FAST)[25] and Strengthening Families 10–14 (SFP 10–14) [26].

Each LA determined how to implement PEIP, including the infrastructure set up to manage and support the parenting courses they provided. Training of group facilitators was organised nationally by NAPP, with training places purchased from the accredited parenting programme provider, but each LA determined the selection of staff to be trained. As with the Pathfinder, the government required LAs to target parents of 8–13 year old children exhibiting or at risk of developing behavioural difficulties.

Local authorities had substantial autonomy as part of government policy and this was increased over the period of the PEIP. They were free to select any one or more of the five accredited parenting programmes to implement; this number later increased to eight following evaluation of further programmes by NAPP.

Initially, LAs were required to provide data to the DfE on the numbers of parents enrolled on PEIP-funded parenting courses. This policy was not maintained, however, as a consequence of which there are no official data on the numbers of parents across all LAs in England who received parenting programmes through PEIP.

### The programmes

Five parenting programmes were used throughout Waves 2 and 3 of the PEIP, the focus of the present paper: the three originally used in Wave 1 (the Pathfinder): Triple P (*n*=3171 parents, 51.6% of all parents in Waves 2 and 3); Strengthening Families Strengthening Communities (SFSC) (*n* = 868, 14.1%), and the Incredible Years (*n* = 782, 12.7%); together with two programmes introduced in Waves 2 and 3: Strengthening Families Programme 10–14 (SFP 10–14) (*n* = 969, 15.8% of all parents in Waves 2 and 3) and Families and Schools Together (FAST), (*n* = 104, 1.7%). Together these five programmes accounted for 95.1% of all parents (*n* = 5894 in Waves 2 and 3). Later in Wave 2, three other programmes were judged by NAPP to have met the criteria for inclusion: Parent Power (*n* = 104, 1.7% of all parents in Waves 2 and 3; STOP (*n* = 102, 1.7%), and Parents Plus, (*n* = 102, 1.7%) [12].

Evidence for the Pathfinder (Wave 1) programmes has been reviewed in Lindsay, Strand and Davis [2]. Triple P and Incredible Years have been judged by the United Nations Office on Drugs and Crime [11] to be the parenting programmes with the best levels of scientific evidence, rated first and second respectively. FAST and SFP 10–14 are also rated in the UNODC list of the top 23 parenting programmes, 11^th^ and 16^th^ respectively. The other four programmes are not included in the UNODC list but were reviewed and approved by NAPP, see [12] for evidence.

All programmes are manualised with detailed training programmes for group facilitators and specification for supervision of facilitators after they begin to lead groups (see [2] for more details). Triple P (Level 4 was evaluated in the present study) comprised five two-hour face-to-face sessions and three on the telephone (11.5 h); SFSC comprised 12 three-hour group sessions (36 h); Incredible Years, 17 two-hour sessions (34 h); SFP 10–14, 7 two-hour sessions (14 h) plus 4 optional booster sessions 6–12 months after the initial programme; and FAST comprised 8 two-and-a-half hour sessions (20 h), with recommended follow-up monthly booster sessions (2.5 to 4 h) led by parents who have completed this programme. The five main programmes therefore, differed in length and both SFP 10–14 and FAST included families, not just parents, and were always school-based, whereas the other programmes were held in a variety of community and health settings. All programmes ran weekly except for holiday periods. FAST may run for up to about 40 parents, arranged in family groups within the class whereas the other four programmes aimed for classes of about 10.

### Participants

#### Local authorities

The data reported here are drawn from the Parenting Early Intervention Programme (PEIP: 2008–11); data from the Pathfinder are described by Lindsay et al. [2] but included here for reference, as appropriate. A sample of 47 LAs was selected from the 152 English LAs. All 23 Wave 2 LAs were included, a sample of 24 Wave 3 LAs were selected to ensure that the total sample was representative of the range of LAs in England with respect to size, urban and rural, ethnicity, and levels of social disadvantage. Four LAs did not engage sufficiently with the study to provide data, resulting in a total sample of 43 LAs. These four LAs had difficulty in setting up and implementing the PEIP, in terms of creating an appropriate organisational infrastructure and, as a result, had problems implementing a programme of parenting classes.

#### Parents

The parents (*N* = 6143: 85.4% female, 14.6% male) were recruited locally by a range of methods. Some LAs created new or developed existing services to organise parenting classes in collaboration with the parenting programme providers. Other LAs commissioned organisations, including those in the voluntary and community sector, to take on this role.

Table 1 presents the demographic characteristics of parents and target children at the start of their parenting class for the total sample in Waves 2 and 3. The majority of parents (90.6%) were biological parents, predominantly White British (80.7%). There was a substantial minority of Asian heritage (8.3%), with similar proportions whose heritage was Pakistani (3.0%), Indian (2.2%) or Bangladeshi (2.2%). Most parents were socially disadvantaged: 53.8% had left school at the end of compulsory education in England (16 years) with fewer than five passes in the General Certificate of Secondary Education (30.3%) or no qualifications at all (23.5%). However, it is also of interest that a third had attended higher education: 11.3% had a university degree and a further 19.6% had higher education qualifications below a degree level, indicating the heterogeneity among the overall sample of parents attending the parenting classes.

**Table 1:** Descriptive statistics for parent and child background measures for Waves 2 and 3

**Variable**	**Value**	***N***	**%**
Parent Gender	Male	888	14.6
(*n = 6095)*	Female	5207	85.4
Relationship to child	Biological parent	5413	90.6
(*n* = 5977)	Step parent	147	2.5
	Parent’s partner (living together)	141	2.4
	Adoptive parent	59	1.0
	Foster parent	36	0.6
	Other	181	3.0
Family structure	Single parent	2580	43.9
(*n* = 5879)	Living with partner or other adult	3299	56.1
Parent ethnic group	White British	4840	80.7
(*n* = 5995)	White other group	152	2.6
	Mixed heritage	161	2.7
	Asian	498	8.3
	Black	256	4.3
	Any other ethnic group	88	1.5
Parent’s highest	None	1327	23.5
Qualification	Fewer than 5 GCSEs	1707	30.3
(*n* = 5636)	5 or more GCSE at A* - C	546	9.7
	A/AS level	312	5.5
	Higher education below degree	1106	19.6
	Degree	638	11.3
Housing	Own property	1873	32.1
(*n* = 5838)	Rented property	3653	62.6
	Other	312	5.3
Parent health	Family doctor	3010	49.0
(*n* = 4253)	Psychiatrist	525	8.5
	Counsellor	910	14.8
	Social worker	1312	21.4
	Other professional	1456	23.7
Child gender	Male	3539	61.3
(*n* = 5773)	Female	2234	38.7
Child age	Mean (*SD*)	8.6 years	(3.9)
Completed post-test			50.8%
Total sample		6143	

Note. A* is the highest grade at GCSE national examinations.

A high proportion (43.9%) was living as single parents and 62.6% were living in rented accommodation. There was also a high level of engagement with health and social services (69.2%), most commonly with the family doctor (49.0%) but also a social worker (21.4%), counsellor (14.8%), psychologist (8.5%), or other professional (23.7%) which included psychologists, community workers, health visitors, teachers, family support workers, occupational therapists, police, and child behaviour support teams.

#### Children

Each parent was asked to select the child whose behavioural, emotional and social development gave them most concern to be the target child for the purpose of the study.

The majority of the target children were boys (61.3%) compared to 38.7% girls. The mean age was 8 years 6 months (*SD* = 3 years 9 months). Only about half (53.8%) were in the target age for PEIP with 37.2% aged 7 years or less but only 9.1% aged 14 years or more.

Almost half (48.9%) of the children were eligible for a free school meal (FSM) compared to national average of 16% [27], further demonstrating the high proportion of socioeconomically disadvantaged families in the sample. Furthermore, a large proportion of the children had a statement of special educational needs (11.5% compared with 2.7% nationally [28] and almost a third (31.7%) were receiving additional educational support, including: one-to-one additional support at school, anger management, booster classes, counselling, home tutoring, and focussed educational support for dyslexia and other learning difficulties. Additional support was provided by teaching assistants, learning mentors and nurture classes or groups.

#### Waves 2 and 3 compared with Wave 1

Comparison of the present Waves 2 and 3 sample with the Wave 1 (Pathfinder) sample indicates that the demographic profile was very similar for both parents and target children on the measures that were common in both studies (Table 2). The relative proportions for female participants (Wave 1, 86.7%; Waves 2 and 3, 85.4%) and relationship with the child (e.g. 91.2% vs 90.6% biological parent) and the proportion of boys (Wave 1: 62.3%; Waves 2 and 3: 61.3%) were highly similar. The measure of educational experience was changed for the present study so data are not directly comparable; however in each case about half of the parents had either left school by the age of 16 years or earlier (Wave 1: 48.9%) or had passed fewer than 5 GCSEs at grades A* - C (Waves 2 and 3: 53.8%). The percentage of parents who had sought help from a professional over the previous 6 months was similar in Wave 1 (66.6%) and Waves 2 and 3 (64.2%). There was a small difference with ethnicity, with 80.7% White British parents in Wave 2/3 (close to the national average from the 2011 census of 80.5%) versus a slight under-representation of White British parents (76.1%) in Wave 1. The only difference of real note was that the mean age of the children for Waves 2 and 3 was slightly lower (8.6 years) compared with 9.3 years in Wave 1.

**Table 2:** Descriptive statistics for parental and child background measures for Wave 1 compared with Waves 2 and 3

		**Wave 1**		**Waves 2 and 3**	
**Variable**	**Value**	***N***	**%**	***N***	**%**
**Parent Gender**	Male	291	13.3	888	14.6
	Female	1903	86.7	5207	85.4
**Relationship to child**	Biological parent	1916	91.2	5413	90.6
	Step parent	54	2.6	147	2.5
	Parent’s partner	39	1.9	141	2.4
	Adoptive parent	12	0.6	59	1.0
	Foster parent	30	1.4	36	0.6
	Other	51	2.4	181	3.0
**Parent ethnic group**	White British	1471	76.1	4840	80.7
	White other group	72	3.7	117	2.6
	Mixed heritage	50	2.6	161	2.7
	Asian	229	11.9	491	8.2
	Black	69	3.6	256	43
	Any other ethnic group	41	2.1	95	1.6
**Parent highest level of education**	Left school at 16 or earlier	941	48.9		
	Left school at 17 or 18	220	11.0		
	FE college/apprenticeship	580	28.9		
	Attended university	267	13.3		
**Parent highest qualification**	None			1327	23.5
	Fewer than 5 GCSEs			1707	30.3
	5 or more GCSEs at A*- C			546	9.7
	A/AS level			312	5.5
	Higher education below degree level			1106	19.6
	Degree			638	11.3
**Parent health**	Family doctor	1107	59.0	3010	49.0
	Social worker	373	19.9	1312	21.4
	Counsellor	321	17.1	910	14.8
	Psychiatrist	146	7.8	525	8.5
	Other professional	432	23.1	1456	23.7
	At least one of the above	1469	66.6	4253	69.2
**Child gender**	Male	1247	62.3	3539	61.3
	Female	755	37.7	2234	38.7
**Child age**	Mean (*SD*)	9.3	(3.2)	8.6	(3.9)
**Completed post-test**			50.8%		54.0%
**Total sample**		2207		6143	

A* is the highest grade at GCSE national examinations.

### Intervention

Each programme was manualised and the facilitators leading the classes were trained in the administration of that specific programme by the parenting programme providers themselves. In some cases, LAs trained facilitators in more than one programme in order to provide a range of parenting programmes for parents to access. Twenty LAs implemented a single programme, 11 ran two programmes, 10 ran three programmes, and two ran four or more. Where an LA ran multiple programmes it was usual for one to predominate. Also, some LAs had trained some facilitators prior to PEIP. The DfE did not require LAs to record the numbers of facilitators trained, but as part of the current study we surveyed facilitators running the classes, a total of 1277 (89.4% female, 10.6% male) in the 43 LAs. The largest percentage (37.1%) was aged 40–49 years, although there were also 24.3% aged 30–39 and 21.7% aged 50–59. Facilitators had a range of professional backgrounds including education, nursery qualification, social work, health and community or therapy.

The LA senior staff managing the PEIP, or their counterparts where implementation was carried out by another organisation commissioned for this purpose, provided support, supervision and follow up checks on the fidelity of programme implementation. This was supplemented by the usual follow up by programme staff, to optimise fidelity of implementation and the development of facilitators’ skills. The parenting programmes were delivered in a range of community settings, representative of the locations usually used for these programmes, including schools, health settings, community centres, and voluntary bodies’ premises.

The parenting classes were implemented according to the approach of each programme. This was generally delivered to groups of about 10 parents, except for the FAST programme which is designed to engage larger numbers of parents attending each course. The facilitators ran the courses in accord with that programme’s manual. This included watching DVDs to stimulate discussion of particular teaching points. Role play and discussion sessions were also used. The FAST programme also comprised additional approaches including parents making a meal for others in their group within the larger class. Personal support materials were provided and homework was also carried out.

### Outcome measures

The measures used were generally identical to those used in Wave 1 (Pathfinder): the Parenting Scale-Adolescent [29], the Warwick-Edinburgh Mental Well-being Scale [30] and the Strengths and Difficulties Questionnaire (SDQ) [31]. The only difference was the reduction in number of measures of parenting from four to two: by omitting the Being a Parent scale [32] which measures Satisfaction with being a parent and Parenting Efficacy, in order to reduce the demands on parents. All measures had originally been selected to measure domains where improvements might be expected following parents’ attendance at any of the parenting programmes accredited for the PEIP.

#### Parenting style

The Parenting Scale – Adolescent [29] is a shortened form of a 30 item scale of the same name [33] comprising 13 items, each scored on a 7 point scale. It comprises two subscales each of six items, Laxness and Over-reactivity (each with a range of 6 – 42), together with a single item, Monitoring, which are aggregated to produce a Total Score (range 13–91). Both scales had good reliability (internal consistency) with Cronbach’s alpha .78 for Laxness and .74 for Over-reactivity. High scores indicate less effective parenting styles.

#### Parent mental well-being

Parent mental well–being is a protective factor for child outcomes. The Warwick-Edinburgh Mental Well-Being Scale (WEMWBS) [30] provides a measure of mental well-being as parents started their group and again on completion. It comprises 14 items scored from 1 (none of the time) to 5 (all of the time). High scores represent greater mental well-being. Reliability (internal consistency) was high (Cronbach’s alpha .93). The national median score is 51 (Inter-quartile range 45–56).

#### Child behaviour

Parents rated the behaviour of their target child on the Strengths and Difficulties Questionnaire (SDQ) [31]. The SDQ comprises four scales representing different problem domains: Emotional Symptoms, Conduct Problems, Hyperactivity, and Peer Problems. These four scales are summed to produce a Total Difficulties scale. In addition, a Prosocial scale measures positive behaviours and an Impact scale measures the extent of the impact of the child’s behavioural difficulties on the family, school and wider community. Each scale comprises five items scored on a 3 point scale (0 = not true, 1 = somewhat true, 2 = certainly true, range 0 – 10). Total Difficulties range is 0 – 40 with high scores representing greater difficulties; for the Prosocial scale low scores represent greater difficulties.

The SDQ has been used extensively and has very good evidence of its factor structure, reliability and validity [34]. British norms are available for children 5 – 15 years [35]. Reliability was satisfactory in the present study: internal consistency (Cronbach’s alpha) was .70 or greater for all scales except peer problems (.62); Total Difficulties was .83.

### Procedure

Parents completed the questionnaires which were then returned to the research team for analysis, during or just prior to the first session (pre- course) and again during the last session of their parenting programme (post-course). One year after completing their programme, parents that had completed the post-course questionnaire (N = 3325) were sent the same three questionnaires by post, together with a reply paid envelope in order to examine the persistence of any improvements made between the pre- and post- test measures. If no response was received from a parent within a month, a second, reminder letter was sent.

### Ethical approval

Ethical approval was given by the University of Warwick Humanities and Social Sciences Research Ethics Committee (Ref: Eth App 45/07-08). Parents were provided with information about the study and gave informed consent for their involvement. They were informed that all data would be anonymised and that they were free to withdraw at any time and have their data removed.

### Analysis

Of the 6143 parents who completed pre-course booklets, 3325 (53.5%) returned post course booklets. Results were very similar across the programmes: 52.2% Triple P, 56.8% Incredible Years, 54.1% SFSC, and 58.3% for SFP 10–14. Facilitators were asked to report on the reason for parent non-completion of post- course booklets. For 16% of parents the facilitator provided a reason for non-completion by the parent, which included (a) the parent did not complete the course or only completed a limited number of sessions (12%); (b) the parent completed the course but declined to complete the booklets (1%) or (c) the parent did not complete the booklet for some other reason (2%) (e.g. transferred to another group, moved away from the area, parent ill health, sick child, or gained employment). For a large proportion of parents (32%) there was no facilitator’s report from the group, so reasons for non-completion are not known. However this reflects issues of administrative error and non-response at the group rather than individual level, since of the 860 PEIP groups that were run there were 181 groups where no post-course booklets at all and no facilitator form was returned. This suggests a substantial proportion of the non-response is due to administrative issues at the group level rather than parent drop-out.

A key issue revolves around whether those who responded to the post-course questionnaire differ systematically from those who did not respond. There were some differences between post-course non-responders and responders in demographic characteristics. Those who did not respond at post-course were more likely to be socio-economically disadvantaged than responders: specifically they were more likely to have no educational qualifications (28% vs. 20%, *p < .*001); less likely to own their own property (28% vs. 35%, *p < .*001), more likely to have a child entitled to a free school meal (FSM 52% vs. 47%, *p < .*001), and more likely to be from single parent families (47% vs. 42%, *p* < .001). There were no significant differences in terms of parent gender or ethnicity; or in terms of child age, gender or SEN. However it is differences in pre-course scores that are most salient to the comparison. The pre-course scores for those who did not respond at post-course ('pre-course only’) were compared with those who did respond ('pre & post course’). There were only two significant differences (*p* <.01) indicating those who did not respond at post-course were more likely to have lower mental well-being and higher parenting laxness scores at the start of the course than those who did respond. However given the sample size of over 6,000, statistical significance is a poor guide since small or even trivial differences are likely to be statistically significant. Evaluation of effect size indicates difference between responders vs. non-responders were minimal; for example even the largest difference, that for mental well-being score, was less than one score point on a measure with a SD of over 10 points. In sum there is little evidence that non-responders at post-course differed substantially from those that did respond in terms of pre-course scores.

Consequently the analysis focuses on parents with complete data, calculating change scores (post-test score minus pre-course score) for all scales to give measures of improvement. When making comparisons between programmes ANOVA was employed to control for differences between programmes in demographic variables. This method is preferable to an Intention to Treat (ITT) analysis when there is a substantial loss of participants with post-test scores (for a discussion of the rationale for ANOVA rather than ANCOVA: see Lindsay et al., 2011, p6) [2]. Furthermore, as the percentages of parents with both pre- and post-test scores were so similar across programmes, there is no reason to expect that an ITT analysis would change the estimates of relative programme effects: an ITT analysis would downgrade the effect scores to a similar extent owing to the loss of participants.

## Results

### Outcomes in Waves 2 and 3

#### Parents

In this section we begin by reporting the results for the total sample of parents in Waves 2 and 3. Second, we compare the results for the Waves 2 and 3 sample with Wave 1 (Pathfinder, see Lindsay et al. [2]). Third, we present the results of a one year follow up of Waves 2 and 3 parents. Fourth, we compare the results from different programmes used in Waves 2 and 3. Finally, we present the results for children aged 8-13 years.

There were substantial improvements on all parent measures: *p* < .001 in all cases (Table 3). When they started their programme, parents’ mental well-being was low. Approximately 75% of PEIP parents scored below the national median: PEIP median 43 (inter-quartile range 36 to 51), national median 51 (inter-quartile range 45 – 56).

**Table 3:** **Parenting behaviours: Pre- and post-course means ( *SDs *) and effect sizes of improvements for Wave 1 and for Waves 2 and 3 combined**

	**Wave 1**				**Waves 2 and 3**			
	***N***	**Precourse**	**Postcourse**	**Effect**	***N***	**Precourse**	**Postcourse**	**Effect**
		**(**^**a**^**Mean, *SD*)**	**(**^**a**^**Mean, *SD*)**	**size**		**(**^**a**^**Mean, *SD*)**	**(**^**a**^**Mean, *SD*)**	**size**
Mental well-being	1071	43.5	50.6	0.71	3160	43.5	51.6	0.79
		(10.4)	(9.8)			(10.8)	(9.5)	
Parenting laxness	1040	22.0	17.4	- 0.71	3125	21.3	16.5	- 0.72
		(6.6)	(6.3)			(7.2)	(6.5)	
Parenting over-reactivity	1032	22.5	17.4	- 0.83	3140	22.5	16.9	- 0.85
		(6.4)	(6.2)			(6.9)	(6.4)	
Parenting total score	1030	47.4	37.1	- 0.91	3093	46.6	35.7	- 0.91
		(11.1)	(11.6)			(12.1)	(11.9)	

Note. Effect sizes are Cohen’s *d.*

^a^ The median is reported for mental well-being.

Table 3 presents the mean and standard deviation of each measure and the effect size (Cohen’s *d*) or pre-post comparison, where 0.2 is a small effect, 0.5 a medium effect and 0.8 a large effect. There was a substantial improvement in mental well-being; pre-course *M* = 43.5 (*SD* = 10.8), post-course *M* = 51.6 (*SD* = 9.5), *d* = 0.79. There were substantial reductions in all measures of parenting style: Laxness *d* = -0.72; Over-reactivity, *d* = -0.85; and Total Score, *d* = -0.91. These improvements indicate medium to large effects and suggest that after attending a parenting programme parents were less likely to give in inappropriately to their child or overreact when their child displayed behaviour difficulties.

#### Child behaviour

At the start of their programme a high proportion of parents rated their child’s behaviour in the clinical range on the SDQ, compared with the norm of about 10 per cent on each measure: Conduct Problems, 62.2%; Total Difficulties, 56.7%; and Impact, 61.2%. Overall, there were consistent, statistically highly significant (*p* < .001) improvements as indicated by reduction in mean scores, except for the Prosocial scale which has a reverse scoring: increase in score indicates improvement. The overall level of improvement, as indicated by the effect sizes, was lower than for the parent measures. Close examination indicates that the largest improvements were in Conduct Problems (*d* = -0.45), Total Difficulties (*d* = -0.45) and Impact (*d* = -0.52) – see Table 4. By contrast, improvements in the Peer Problems measure, although also highly significant (*p* < .001) had a small effect size (*d* = -0.15).

**Table 4:** **Child’s behaviours: Pre- and post-course means ( *SDs *) and effect sizes for Wave 1 and for Waves 2 and 3 combined**

	**Wave 1**				**Waves 2 and 3**			
	***N***	**Precourse**	**Postcourse**	**Effect**	***N***	**Precourse**	**Postcourse**	**Effect**
		**(Mean, *SD*)**	**(Mean, *SD*)**	**size**		**(Mean, *SD*)**	**(Mean, *SD*)**	**size**
Emotional symptoms	1067	3.8	2.8	- 0.42	3154	3.8	2.9	- 0.37
		(2.5)	(2.3)			(2.6)	(2.5)	
Conduct problems	1071	4.3	3.1	- 0.55	3144	4.4	3.4	- 0.45
		(2.4)	(2.2)			(2.5)	(2.3)	
Hyperactivity	1053	6.2	5.0	- 0.43	3138	6.2	5.3	- 0.34
		(2.7)	(2.6)			(2.7)	(2.7)	
Peer problems	1064	3.3	2.8	- 0.24	3131	3.3	3.0	- 0.15
		(2.2)	(2.1)			(2.3)	(2.2)	
Prosocial	1068	6.4	7.0	- 0.26	3165	6.4	6.9	0.22
		(2.3)	(2.1)			(2.3)	(2.2)	
Total difficulties	1038	17.5	13.5	- 0.57	3028	17.8	14.5	- 0.45
		(6.9)	(7.0)			(7.2)	(7.4)	
Impact	1031	2.9	1.7	- 0.48	3046	3.0	1.7	- 0.52
		(2.7)	(2.4)			(2.8)	(2.4)	

Note. Effect sizes are Cohen’s *d*.

The SDQ also has norms to categorise children’s scores according to a 3 category system: 10% abnormal, 10% borderline and 80% normal [32]. The percentage of children rated by their parents as having clinically significant ('abnormal’) difficulties reduced by a third: Conduct Problems decreased from 61% to 41%, Total Difficulties from 57% to 38% and Impact decreased even more, from 62% to 36%.

### Comparison of Waves 2 and 3 with Wave 1 (Pathfinder)

As indicated in Table 1, the demographic profiles were very similar for Wave 1 compared with Waves 2 and 3. The outcomes for Waves 2 and 3 from 43 LAs were also very similar to those of the Wave 1 LAs, for both parents’ and children’s scores on all measures, with respect to means (*SDs*) and effect sizes. For example, mean parent Mental Well-Being improved from 43.5 to 50.6 Wave 1 compared with an improvement from 43.5 to 51.6 for Waves 2 and 3; effect sizes were also similar: 0.71 and 0.79 respectively (Table 3). Mean Parenting Scale scores decreased (indicating less laxness and over-reactivity) from 47.4 to 37.1 (Laxness) and from 46.6 to 35.7 for Over-reactivity with effect sizes -0.91 in each case.

The effect sizes of improvements in parents’ ratings of their children’s behaviour were at a lower level for Waves 2 and 3: Conduct Problems: -0.55 Wave 1, -0.45 Waves 2 and 3; and for Total Difficulties: -0.57 Wave 1, -0.45 Waves 2 and 3 (Table 4). However, Impact showed the opposite trend: Wave 1 effect size -0.48, Waves 2 and 3 effect size -0.52.

### One year follow up

The response rate to the one year follow up was 1260 (37.9%) of the 3325 parents who were in the sample, comprising 1035 (30.5%) parents that completed booklets of questionnaires and 225 (6.6%) where the booklet was returned but indicating that the addressee had moved away. High mobility was an important factor for PEIP parents, with two thirds living in rented accommodation compared to about a quarter nationally (see Table 1). The parents for whom we received valid data were drawn from all 43 LAs and comprised parents that had attended one of the four main PEIP programmes: Triple P *n* = 473 (45.7%); Incredible Years *n* = 146 (14.1%); SFSC *n* = 169 (16.3%); and SFP 10–14, *n* = 176 (17.0%). In addition, 71 parents (6.9%) had attended one of the other four programmes.

A series of one-way repeated measures ANOVAs was conducted to examine the effect of occasion: pre-course, post-course and one year follow-up, based on cases with valid scores at all three time points. Pairwise comparisons with Bonferroni correction were conducted as post hoc tests. Means (*SDs*) are presented in Table 5.

**Table 5:** **Parenting and child measures (*M, SD) *** **at pre-course, post-course and one year follow up**

	**Pre-course**		**Post-course**		**Follow up**	
	***M***	***SD***	***M***	***SD***	***M***	***SD***
***Parents***						
Mental well-being	43.9	10.6	51.8	9.3	49.1	11.3
Parenting laxness	20.5	7.3	15.6	6.1	16.8	6.2
Parenting over-reactivity	22.5	6.8	16.8	6.3	17.9	6.3
Parenting total score	45.7	12.1	34.6	11.3	37.0	11.6
***Child***						
SDQ total difficulties	17.5	7.5	14.2	7.7	14.0	8.2
SDQ conduct problems	4.3	2.5	3.2	2.3	3.3	2.5

Note. Based on parents with valid scores on all three occasions, specifically: 923, 914, 912, and 901 parents respectively for mental well-being, laxness, over-reactivity and parenting total scores; and 864 and 890 parents respectively for child total difficulties and conduct problems.

#### Parent outcomes

##### Mental well-being

**Figure 1:**
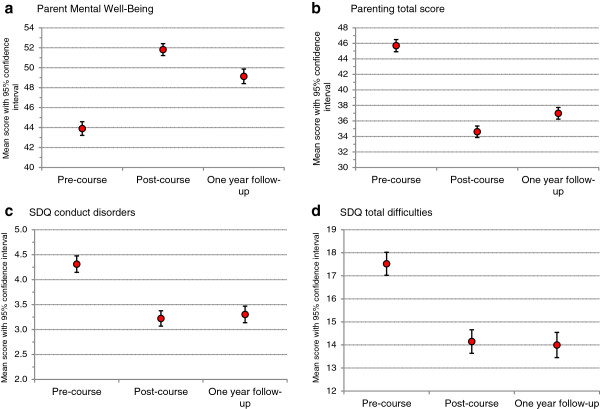
Pre-, post-course, and one year follow up means with 95% confidence intervals: (a) parent mental well-being; (b) parenting style total score; and child total difficulties (total sample).

There was a highly significant effect of occasion: *F*(2, 1844) = 295.4, *p* < .001, partial eta squared = .24). Pairwise comparisons of means with Bonferroni correction indicated that parent mental well-being at post-course was significantly higher than at the pre-course (*p* < .001)*.* The mean score at the one year follow-up fell back relative to the immediate post-course score (by 2.7 points) but remained significantly higher than at pre-course (by 5.2 points, *p* < .001) (see Figure 1a).

##### Parenting behaviours: laxness and over-reactivity

There was also a highly significant effect for both parenting measures: Laxness, *F*(2, 1826) = 270.5, *p* < .001, partial eta squared = .30; Over-reactivity, *F*(2, 1822) = 367.3, *p* < .001, partial eta squared = .29. The Parenting total score followed this pattern but with a greater effect size: *F*(2, 1800) = 448.1, *p* < .001, partial eta squared = .33. Pairwise comparisons of means with Bonferroni correction indicated significant reductions in the negative parenting behaviours of laxness and over-reactivity at immediate post-course (*p* < .001). Scores increased slightly at one year follow up, but remained significantly below pre-course levels (*p* < .001) (see Figure 1b).

##### Child behaviour

**Figure 2:**
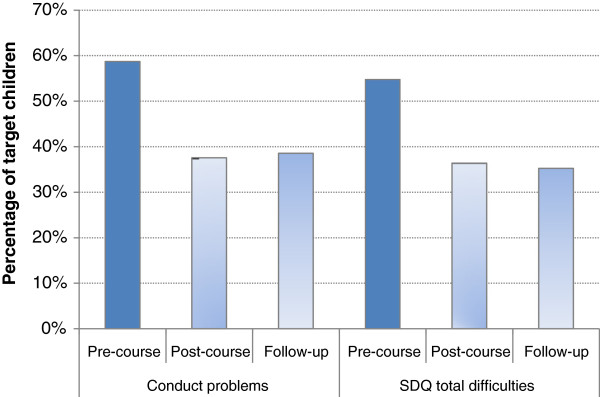
Percentage of target children rated in the clinical range for conduct problems and total difficulties at pre-course, post-course and one year follow-up (total sample).

A highly significant effect of occasion was found for child SDQ total difficulties: *F*(2, 1726) = 212.9, *p* < .001, partial eta squared = .20; and conduct problems *F*(2,1778) = 157.6, *p* < .001, partial eta squared = .15). Pairwise comparisons of means with Bonferroni corrections indicated significant reductions in child total difficulties and conduct problems at post-course (*p* < .001) which were maintained at follow up, with no significant differences between post-course and one year follow up scores. In terms of the classification of the child’s difficulties in the clinically significant ('abnormal’) range, the pattern was similar (Figure 2).

### Programme differences in patterns of change over time

In this section we examine whether there were any statistically significant differences between the programmes with respect to improvements on the parent and child measures. The analyses of programme effectiveness include only the four programmes with substantial numbers of parents which together accounted for 93% of all parents in Waves 2 and 3: Incredible Years; Strengthening Families 10–14 (SF 10–14); Strengthening Families Strengthening Children (SFSC); and Triple P. As the largest programme, Triple P was set as the base programme against which the other programmes were compared.

There were small differences between programmes in the demographic profile of their parents and children in terms of the parent and child background variables. The analyses controlled for these differences by estimating programme effects after accounting for variation associated with all significant parent and child background variables. These models included only those parent and child background variables that were significantly associated with each outcome. The models also included fixed effects for each of the 43 LAs to control for variation between LAs in outcomes.

All four programmes were effective as shown in Table 6. Effect sizes for parenting measures were medium to large: for example, ranging from 0.66 for SFP 10 – 14 to 0.83 for Triple P and Incredible Years for mental well-being. The pattern was comparable to that of the total sample with higher effect sizes for the measures of parenting (medium to large) than child behaviour, which were small to medium in size, for all programmes.

**Table 6:** Improvements in parenting and child behaviour between pre-course and post-course for Incredible Years, Strengthening Families Programme 10-14, Strengthening Families Strengthening Communities, and Triple P

		**Incredible years**				**SFP 10-4**				**SFSC**				**Triple P**			
	**Variable**	**N**	**Pre- course**	**Post- course**	**Effect size**	**N**	**Pre- course**	**Post- course**	**Effect size**	**N**	**Pre- course**	**Post- course**	**Effect size**	**N**	**Pre- course**	**Post- course**	**Effect size**
**Parent**	Mental Well-Being	431	43.9	52.1	**0.85**	539	40.7	47.5	**0.69**	453	44.0	51.6	**0.74**	1562	44.1	52.7	**0.85**
			(10.1)	(9.3)			(10.6)	(9.4)			(10.9)	(9.4)			(10.8)	(9.3)	
	Parental laxness	415	20.9	16.0	**-0.73**	533	22.1	18.2	**-0.57**	439	20.8	17.1	**-0.54**	1563	21.5	15.9	**-0.83**
			(6.9)	(6.4)			(7.3)	(6.5)			(7.2)	(6.6)			(7.2)	(6.3)	
	Parental overreactivity	418	22.1	16.9	**-0.77**	538	24.1	19.3	**-0.74**	438	21.8	17.4	**-0.71**	1571	22.4	16.0	**-0.96**
			(7)	(6.3)			(6.5)	(6.4)			(6.5)	(6.2)			(6.9)	(6.3)	
	Parenting total score	407	45.5	35.2	**-0.86**	529	49.3	40.0	**-0.79**	432	45.3	36.9	**-0.71**	1554	46.6	34.1	**-1.04**
			(12)	(11.8)			(11.8)	(11.8)			(11.7)	(11.7)			(12.2)	(11.7)	
**Child**	Emotional symptoms	424	3.8	2.9	**-0.36**	547	4.6	3.7	**-0.36**	437	3.6	2.7	**-0.37**	1565	3.6	2.7	**-0.40**
			(2.6)	(2.5)			(2.8)	(2.7)			(2.6)	(2.5)			(2.6)	(2.4)	
	Conduct problems	425	4.2	3.2	**-0.42**	545	5.2	4.2	**-0.41**	433	4.0	3.3	**-0.32**	1559	4.4	3.2	**-0.54**
			(2.4)	(2.4)			(2.5)	(2.5)			(2.4)	(2.3)			(2.4)	(2.2)	
	Hyperactivity	424	6.1	5.2	**-0.33**	547	6.4	5.7	**-0.26**	427	6.0	5.3	**-0.25**	1562	6.3	5.2	**-0.40**
			(2.8)	(2.8)			(2.7)	(2.7)			(2.7)	(2.6)			(2.7)	(2.6)	
	Peer problems	420	3.1	2.9	**-0.10**	545	3.7	3.5	**-0.11**	427	3.2	2.9	**-0.17**	1560	3.3	2.9	**-0.17**
			(2.2)	(2.1)			(2.4)	(2.3)			(2.2)	(2)			(2.2)	(2.1)	
	Prosocial scale	427	6.6	6.9	**0.17**	546	6.4	6.6	**0.13**	430	6.3	6.8	**0.24**	1584	6.4	7.0	**0.27**
			(2.1)	(2.1)			(2.3)	(2.3)			(2.2)	(2.2)			(2.3)	(2.1)	
	Impact	413	2.8	1.6	**-0.47**	531	4.2	2.5	**-0.61**	426	2.5	1.5	**-0.37**	1500	2.9	1.4	**-0.57**
			(2.7)	(2.3)			(2.9)	(2.7)			(2.8)	(2.4)			(2.8)	(2.3)	
	SDQ total score	411	17.3	14.1	**-0.43**	532	20.0	17.1	**-0.40**	413	16.8	14.0	**-0.39**	1496	17.6	14.0	**-0.51**
			(7.1)	(7.6)			(7.2)	(7.6)			(7.2)	(7.3)			(7)	(7.1)	

Note. Effect sizes are in bold to aid clarity.

For parent mental well-being and parenting laxness, SFP 10–14 was less effective than Triple P, but no other differences were statistically significant. For parenting over-reactivity both SFP 10–14 and Incredible Years were less effective than Triple P, but no other differences were statistically significant. With respect to child outcomes, there were no differences between programmes for child total difficulties or for impact. However Triple P was significantly better than the other three programmes at reducing reported child conduct problems.

It is notable however that even after including parent demographics, programme type and LA, the *R*^2^ for each outcome was never higher than 6.4%^a^. Therefore while these effects are statistically significant they explain only a small proportion of the change in scores. The majority of variance in change relates to individual differences between parents that are not captured in any of the broad parent or child demographics or structural features such as programme type or LA. Some parents were more responsive than others to PEIP, but this reflects unique features of the parents as individuals.

### Children aged 8–13 years

Although the PEIP was aimed at parents of children 8–13 years, a substantial proportion of the children identified by parents as their targeted child were older or younger. We therefore conducted a separate analysis for the 53.8% of parents and children in the target age range. Results were almost identical to those for the total PEIP sample as indicated by Tables 7 and 8, as compared with Tables 5 and 6.

**Table 7:** **Parenting and child measures (Mean, *SD*) at pre-course, post-course and one year follow-up: Parents of children aged 8–13 years only**

	**Pre-course**		**Post-course**		**Follow-up**		**Sample**
	***Mean***	***SD***	***Mean***	***SD***	***Mean***	***SD***	***N***
**Parents**							
Mental well-being	43.9	10.5	51.5	9.6	49.1	11.2	460
Parenting laxness	20.4	7.4	15.9	6.4	17.0	6.2	461
Parenting over-reactivity	23.1	6.7	17.6	6.3	18.3	6.3	460
Parenting total score	46.2	12.0	35.7	11.7	37.7	11.5	455
**Child**							
SDQ total difficulties	18.4	7.6	15.1	7.8	14.9	8.2	456
SDQ conduct problems	4.55	2.52	3.52	2.42	3.63	2.60	465

**Table 8:** Improvements in parenting and child behaviour between pre-course and post-course for Incredible Years, Strengthening Families 10-14, Strengthening Families Strengthening Communities, and Triple P (children aged 8-13 years)

**Age 8–13 only**																	
		**Incredible years**				**SFP 10-14**				**SFSC**				**Triple P**			
	**Variable**	**N**	**Pre- course**	**Post- course**	**Effect size**	**N**	**Pre- course**	**Post- course**	**Effect size**	**N**	**Pre- course**	**Post- course**	**Effect size**	**N**	**Pre- course**	**Post- course**	**Effect size**
**Parent**	Mental Well-Being	200	43.1	50.5	**0.75**	446	41.1	47.3	**0.64**	188	44.0	51.6	**0.71**	634	43.8	52.6	**0.88**
			(10.1)	(9.7)			(10.2)	(9.4)			(11.4)	(9.8)			(10.6)	(9.4)	
	Parental laxness	192	20.8	17.0	**-0.54**	440	21.8	18.3	**-0.52**	186	21.5	17.6	**-0.60**	658	21.3	15.7	**-0.79**
			(7.4)	(6.8)			(7.3)	(6.5)			(6.8)	(6.3)			(7.6)	(6.4)	
	Parental overreactivity	194	22.5	17.9	**-0.72**	442	24.1	19.7	**-0.70**	188	23.5	18.8	**-0.76**	664	23.1	16.2	**-1.06**
			(6.6)	(6.3)			(6.5)	(6.3)			(6.1)	(6.2)			(6.9)	(6.1)	
	Parenting total score	188	46.0	37.3	**-0.71**	436	49.0	40.3	**-0.74**	185	47.8	38.9	**-0.79**	655	47.2	34.3	**-1.09**
			(12.2)	(12.3)			(11.8)	(11.7)			(10.9)	(11.7)			(12.2)	(11.5)	
**Child**	Emotional symptoms	197	4.3	3.1	**-0.45**	453	4.6	3.6	**-0.37**	189	4.3	3.3	**-0.39**	669	4.1	3.1	**-0.40**
			(2.7)	(2.6)			(2.7)	(2.7)			(2.5)	(2.6)			(2.6)	(2.4)	
	Conduct problems	198	4.4	3.7	**-0.30**	451	5.2	4.2	**-0.39**	189	4.4	3.6	**-0.32**	667	4.7	3.5	**-0.53**
			(2.6)	(2.6)			(2.5)	(2.5)			(2.5)	(2.4)			(2.4)	(2.3)	
	Hyperactivity	198	6.2	5.4	**-0.27**	453	6.5	5.8	**-0.27**	187	6.1	5.4	**-0.26**	672	6.4	5.3	**-0.39**
			(2.9)	(2.9)			(2.7)	(2.7)			(2.7)	(2.5)			(2.7)	(2.6)	
	Peer problems	196	3.4	3.3	**-0.05**	452	3.7	3.5	**-0.11**	186	3.6	3.2	**-0.18**	667	3.5	3.1	**-0.15**
			(2.3)	(2.3)			(2.5)	(2.3)			(2.3)	(2.2)			(2.3)	(2.2)	
	Prosocial scale	200	6.5	6.7	**0.10**	452	6.4	6.6	**0.11**	189	6.4	6.9	**0.21**	672	6.5	7.0	**0.24**
			(2.2)	(2.1)			(2.3)	(2.3)			(2.3)	(2.2)			(2.4)	(2.2)	
	Impact	192	3.3	2.0	**-0.49**	437	4.2	2.5	**-0.60**	187	3.2	2.0	**-0.44**	636	3.5	1.9	**-0.62**
			(2.7)	(2.6)			(2.8)	(2.7)			(2.9)	(2.6)			(2.7)	(2.5)	
	SDQ total score	192	18.4	15.4	**-0.38**	441	20.1	17.2	**-0.40**	180	18.4	15.6	**-0.38**	654	18.6	15.0	**-0.51**
			(7.5)	(8.2)			(7.4)	(7.7)			(7.5)	(7.5)			(7.1)	(7.2)	

Effect sizes are in bold to aid clarity.

## Discussion

This study examined the national roll-out of parenting programmes across all 152 local authorities (LAs) in England, the Parenting Early Intervention Programme (PEIP). To the best of our knowledge this is the first study that has examined the effectiveness of a national roll-out of parenting programmes and has included a comparison of relative effectiveness of four different programmes and a long term follow up. Furthermore, we provide evidence on an under-researched population, namely children aged 8–13 years.

The present study was the last of a 3-stage process developed by the UK Government’s Department for Education in England. A literature review established that there were parenting programmes with evidence of efficacy: two of the three selected for the Pathfinder (Triple P and Incredible Years) had evidence from many randomized controlled trials (RCTs) [5,11] including RCTs in England, for example [36]. Strengthening Families Strengthening Communities (SFSC) did not have RCT evidence, but rather evidence from pre- to post-implementation studies. The Pathfinder implemented over two years in 18 LAs demonstrated that these three programmes were all effective, although SFSC showed lower effects than the other two [2].

The present study reports the implementation of evidence-based programmes in all LAs in England and a substantial increase in scale and range, from 18 to all 152 LAs. The evidence shows clearly that the effectiveness demonstrated for the three Pathfinder programmes was maintained and also that a fourth programme, SFP 10–14 achieved similar levels of effectiveness. This applied across all of our measures of parental mental well-being, parenting skills (reducing laxness and over-reactivity); and applied also to child conduct problems, total level of children’s behavioural, emotional and social difficulties, and their impact on the family. Indeed, the effect sizes were very similar in both the present study and the earlier Pathfinder despite a tripling of the number of parents providing pre- and post- parenting programme comparison data.

Furthermore, levels of improvement over the period of the programmes (pre- to post- test) are comparable to those found in smaller scale studies. For example, the results for child behaviour problems for Triple P in the current study were similar to the range found in the meta-analysis of 55 studies by Nowak and Heinrichs [5] for a within-group design: 0.52 – 0.55 in the present study compared with 0.45 – 0.57.

The second aim of the study was to examine the longer term effects of parents’ attendance at one of the parenting programmes. One year after completing their parenting courses, parents’ ratings indicated that the improvements made between the pre- and post-course assessment were maintained. Across all parent measures there were small reductions at follow up compared to immediate post-course scores although, with respect to the children, reported improvements were maintained completely at follow up. Taken together, the results of short term (pre- to post-course) and one year follow up indicate that the PEIP achieved similar patterns of initial improvement and of maintenance of these improvements as found in efficacy trials with these evidence based programmes [36,37]. This was achieved from a roll-out across the whole of England, a very substantial scaling up. The roll-out required the training of at least 1277 facilitators in the 43 LAs to provide the resource to implement the parenting programmes, and implementation in a variety of community settings, organised at local level by each LA.

Furthermore, it is of interest to note that the improvements reported by parents following attendance at an evidence based parenting course were very similar to those achieved during the Pathfinder (Wave 1) in 18 LAs, itself a roll-out on a substantial size [2]. The two studies, therefore, indicate both a replication of the effectiveness of the three parenting programmes examined in the Pathfinder and maintenance of the effects when the scale of delivery was increased substantially.

The third aim was to examine the relative effectiveness of the four programmes for which we had sufficient data. All four were effective with medium to large effect sizes for improvements in parents’ mental well-being and parenting skills, and small to medium effect sizes for SDQ child conduct problems, SDQ total difficulties and SDQ impact. After controlling for differences in demographic background variables and initial scores on the measures, there were generally larger effect sizes for Triple P on parent and child measures, but not all between programme comparisons were significant: SFP 10–14 was less effective than Triple P for parents’ mental well-being and parenting laxness; and both SFP 10–14 and Incredible Years were significantly less effective than Triple P for parenting over-reactivity. With respect to child outcomes, Triple P showed significantly greater effects in improving children’s conduct problems than all three other programmes.

These results are similar to those in the Pathfinder in that Triple P generally showed higher effectiveness in that study also. However, in the Pathfinder SFSC was less effective than both Triple P and Incredible Years for both parenting and child measures. Hence, across the two studies there is consistency in demonstrating a general effectiveness across both parent and child measures for the total sample (all programmes combined) but a generally higher level of effectiveness, though not always statistically significant, for Triple P across the range of parent and child measures.

The results for the group targeted by the PEIP, parents of children aged 8–13 years, were almost identical to those of the complete sample. This indicates that these programmes are suitable for parents of children in this age range as well as those with younger children.

The study was rigorous within the parameters of an effectiveness study of a national roll out of the programmes implemented in community settings across all 152 English LAs, building upon our earlier study of the 18 LAs in the Pathfinder. There were, however, limitations which were essentially the same as the Pathfinder [2]. First, post-course data were available on only about half the original sample. As with the Pathfinder only about half of this loss could be attributed to drop outs, the rest was a result of administrative errors. However, this had a knock on effect on the one year follow up as that only targeted parents with both pre- and post-test data. Second, as a real world study on a national scale, random allocation of LAs to programmes was not possible. Furthermore, government policy had changed subsequent to the Pathfinder to allow funding to be used for any of the accredited parenting programmes. Third, LAs varied in their effectiveness of organising their implementation. For example, the numbers of parents attending a programme varied greatly.

## Conclusions

The present study has demonstrated that evidence based parenting programmes can be successfully rolled out on a national scale. The evaluation of programmes when scaled up is important: small scale efficacy trials form a basis for evidence based practice, showing that an intervention can produce positive effects. Community based trials are needed to demonstrate generalisation of effects to 'real life’ practice. But for public policy determination, effects must also be maintained when scaling up to the level of policy coverage, in this case the whole of England.

Furthermore, the present study and the earlier study of the Pathfinder [2], provide evidence for the impact of research in shaping government policy: this developed from a systematic review of the research literature to an evaluated large scale community based trial and eventually to the implementation and evaluation of the national roll-out. At each point, policy was based on evidence of effectiveness.

## Endnote

^a^Alternative models such as ANCOVA with the pre-course score entered as a covariate give a higher R^2^ (e.g. 25.8% for parent laxness). However this is because in such models the R^2^ represents the variation in the outcome score (or state) that can be explained, rather than the variation in the change or improvement score. With ANOVA the R^2^ is a direct measure of the amount of variation in change that can be accounted for.

## Competing interests

The authors declare they have no competing interest other than: GL has presented at conferences organised by three of the parenting programmes to report the results of the earlier pathfinder study: Triple P (2 conferences, fee), Incredible Years and FAST.

## Authors’ contributions

GL led the research (Principal Investigator) and drafted the manuscript; SS was Co-investigator for the study, participated in the design, conducted the analyses and drafted the Results section. Both authors read and approved the final manuscript.

## Authors’ information

GL is Director of the Centre for Educational Development, Appraisal and Research (CEDAR) at the University of Warwick, England where he is also Professor of Educational Psychology and Special Needs Education. SS is Professor of Education at the Department for Education, University of Oxford. GL is currently leading the evaluation of the UK’s Department of Education funded CANparent trial of universal parenting classes; SS is a co-investigator and leading on the analysis of quantitative data from the trial.

## Pre-publication history

The pre-publication history for this paper can be accessed here:

http://www.biomedcentral.com/1471-2458/13/972/prepub
